# Prevention and Treatment of Approximate Decay

**Published:** 1876-10

**Authors:** A. Warner


### ARTICLE III.

### *Prevention and Treatment of Approximate Decay.*

BY A. WARNER, JR., D. D. S.

Dr. Arthur has written an elaborate work on this subject, and I advise all who have not read it, to do so at their earliest convenience, and to practice, at least a part of his teachings.

We all agree that cleanliness must be observed as the one great preventive, and possibly this is all the hygienic treatment required. In order to do this effectually, the teeth must be temporarily separated in many cases, and to do this with the least annoyance and discomfort to the patient pack cotton between the teeth near their cutting edges, and leave for twenty-four hours or longer. If incisors, where it is difficut to retain cotton, pass a floss silk thread between them up to their necks, then pack the cotton in and tie the silk around it. The silk will hold the cotton, and at the same time bind it tighter and make more compression.

Right here let me say to those who are using rubber to make separations for filling, to lay it aside and use cotton as I have just directed. Your patients will thank you for it, and you will have the pleasure of knowing you are saving them an untold amount of misery and dental inflammation. If the teeth are yielding, they may be separated at once with the wedge of wood and operated upon without any serious inconvenience to the patient. After separation, if you only find the enamel discolored, it may be removed with corundum tape alone. If decomposition has already taken place, and the enamel is chalky, it may be cut away with the chisel, using care not to deform the exterior aspect of the teeth. If it is decayed to the dentine, and not into it, it may be removed by the use of corundum wheels, or any instrument the case requires, that will not injure the teeth or surrounding parts. Cut a V shaped space from the palatine surface at the same time you are removing the decay, so when the teeth return to their position, the approximate surfaces will no longer be in contact except at their exterior margin, and the decay will not be likely to attack the teeth in the same manner again.

Exposed dentine will resist decay nearly as effectually as enamel if it is kept clean. I think we all have had this demonstrated to us, by observing the crowns of teeth that are worn away to the dentine, and in many cases to the pulp, where not the least sign of decomposition could be traced. We also know there is some resistance made by nature through the dental tubuli, against the encroachment of caries in the dentine, and in many cases this resistance is sufficient to entirely check decay.

I do not advise separating all teeth regardless of texture and position as a preventive of decay. Where there is no predisposing cause, and the teeth are of good material, and there is sufficient room to cleanse them, and could see the patient frequently, I would not separate.

The bicuspids and molars require different treatment, to some extent, from the anterior teeth. It is good policy to separate the temporary from the permanent teeth. Make large V shaped spaces, by cutting away from the temporary teeth only. I doubt whether it is profitable in all cases to separate the permanent bicuspids and molars before there is any indication of decay. When they are crowded it may be well to relieve them with a thin file, and polish the surface with pumice stone or oxide of tin. Prepare them in such a way that their surfaces may be kept free from vitiated mucus and foreign matter. The second bicuspids may be cut away on both sides if necessary, thus leaving the first bicuspid free and first molar free on its anterior surface. Without the surfaces between the first and second molar and second and third molars are predisposed to decay, it would be well to let them alone, provided the patient uses care, and has frequent examinations made as to their condition.

Dr. Arthur claims that none except the fissure surfaces need be allowed to decay, provided the dentist has charge of the case from childhood, and fully performs his duty.

This statement I am not prepared to admit; however, I have not practiced his method to an extent that enables me to contradict it. After separating the teeth, use floss silk between them frequently, and brush thoroughly and often enough to keep the surface free from foreign substances. Examine as often as every three months if the patient permits.

I have used one of Bonwill's diamond reamers, inserted in a right angle attachment, for separating, but find it to cut very slow. Though it may be used without giving pain it is extremely disagreeable to most patients. I am sorry I can not recommend it in as high terms as the inventor did; my instrument, I think, is imperfectly made, but it came direct from the inventor, and he pronounced it a good one.

The treatment of approximate decay in the incisor teeth, when the cavities are small, is the same as treating for their prevention, viz: To cut away from the lingual surface, as I have already directed, and bevel so their surfaces may be kept free with the brush. Cavities which have penetrated to the dentine may be removed with disfiguring their external aspect in this manner.

But I will not dwell upon this subject, as every operator will treat the cases as his judgment dictates. Small corundum points, wheels and chisels will be quite as good instruments for this purpose as any yet devised, that have come under my observation. There is a class of operators that I would advise not to separate teeth, neither would I advise them to fill teeth.

Where proximate decay has penetrated the dentine to much extent, the filling process is requisite. I will not speak of filling the ordinary cavities, as this is no place for teaching the primitive principles or ground work of our profession. Hence, pass on to that which is difficult; those cavities whereby dentists earn bread by the "sweat of their brow;" those cavities that try our's, and our patient's en durance, and those cavities upon which we fail oftener than succeed, after much ado.

Let us fill one of these now, while we can make a painless operation, and work without instruments; one will be sufficient. Imagine it in, say, the second inferior molar with a deep crown, distal surface, and extending from the crown to much below the gum; pulp exposed and been aching for days; dens sapientia in close juxtaposition; cavity partially filled with gum, and every thing else correspondingly lovely; small mouth; fractious patient, etc.

Possibly it is better I fill this cavity by imagination as it is much easier done. However we have diagnosed this case and find it advisable, owing to the large exposure and inflamed condition of the pulp, and constitution of the patient, to apply a devitalizing agent. This makes, comparatively, an easy cavity to fill, you say. But suppose we do not devitalize? After chipping away the frail enamel, and carefully removing the debris, foreign matter and decomposed dentine, or as much as thought advisable, we find the pulp badly exposed, and perhaps worse than our diagnosis led us to suppose. After carefully washing out the cavity with luke-warm water and drying as much as possible, we cover the pulp with a thin coating of oxide of zinc mixed with creosote, and place over the oxychloride of zinc. This much is done without the aid of the coffer dam, for the gum is yet in the way, and the dam can not be successfully applied. So far I have only removed the irritating substance

(or the cause of pain) and applied the dressing in a manner to last only for a few days, to allow the pulp a chance to recuperate.

Now prepare the case for the next sitting, by dissecting away that portion of the gum. Next day, if the tooth has not given pain, or to any great extent, change the cotton and keep changing from day to day until the gum is sufficiently removed from the cavity to allow the application of the dam successfully; be sure and use cotton, not rubber; at the expiration of a week, or a month, the dam can be applied. It should be punched for the two bicuspids and three molars, if they are all in the mouth. A piece of rubber six inches square is quite large enough for this or any other case. Every thing being now ready to fill the entire cavity, and finish the operation if we so desire—that is, if the tooth has given no trouble since the first application, and they usually do not—apply the dam, soaped if you like it, on the first bicuspid, and secure it there by ligature; pass it over the remaining teeth and clamp over the third molar; if you haven't a suitable clamp, tie it on. Sometimes it may be secured by passing pins between the teeth, and stretching the dam over them, but the clamp holds the mouth open and gives a better opportunity to operate. Place the mouth piece of Fisk's saliva pump in the mouth, turn on the water, and you will not have any trouble from over flow of saliva. But the dam is not sufficiently secure; it leaks at the cervical margin of the cavity; take floss silk, for it is the best thing for the purpose; teeth do not grow so close together but what silk will pass between them, something that can not be said of the other threads; with this silk, single or double as you prefer, the dam can be drawn down to the margin of the cavity and all moisture excluded. The cavity you know thoroughly prepare for a gold filling. The first capping is removed, and another fac similie inserted; leave as much of the os artificial in the cavity as will not endanger the retention of the gold.

This is a delicate place to insert a wedge, but one properly shaped, with a curve, and made very thin can be pressed between the teeth, that will shield the gum and facilitate matters very much.

Begin the filling where you like and how you choose, only make it solid from the first pellet; with the mouth glass in the left hand, light can be reflected where desired. A matrix may be used, but they are only a disadvantage, and work evil in a majority of these cases.

The hard pluggers can now be used to advantage, Williams' cylinders, ditto. Fill across the cervical portion of the cavity, and next to the walls with unannealed gold, if convenient, thereby gaining the advantage of soft foil, if it has any; bind the filling with cohesive gold. After the cavity is half filled it may be policy to finish the filling at the cervical border while you can see how to do it well, using care not to puncture the dam. The main trouble is now over. Finish the filling with heavy foil, or in any manner desired.

Any approximate cavities may be treated in a similar manner as I have just described, and if properly filled with gold success will generally follow.

I can not vouch for amalgam in a similar case. In the May number of the *Missouri Dental Journal*, may be seen under the heading of "Letters to a Young Dentist," a statement made by an all wise being, a man who has seen (so he says) ten thousand amalgam fillings. Just think of it! And yet he styles himself, *Gold Foil.*

This amalgam manipulator says: "Take ten thousand decayed molar and bicuspid teeth in all stages of decay, from an enamel fissure to an exposed pulp, or ulcerating root; teeth soft and teeth hard; chalky teeth and brittle teeth; let the average dentist plug five thousand with a good amalgam, and the other five thousand with gold; inspect them ten years from date, and you will find that more teeth have been saved with the former than with the latter." This is an assertion he says that "one thousand dentists in the West will bear me out in."

Now this is a nice dish to set before a young dentist. If he is only an ordinary dentist he will of course, diet on amalgam if he wants to save teeth. If the young dentist is a thorough bred, he will order his amalgam with plain soda in the morning, and wash it later in the day with alcohol.

Mr. Gold Foil also tells us how to put in this putty filling; he knows just how to do it; in fact, he understands it so well that I imagine seeing him during his palmy days, meandering the suburban streets and alleys of St. Louis, with a box of glass on his back, and a ball of putty in his hands, crying out, "Do you want any glass put in?"

His next subject will be on dental fees, when it is sincerely hoped that young dentists may get better advice than they got in letter number four.

I say to young dentists to treat approximal cavities with gold or tin foil. Make your failures, meet them face to face and try again. Acknowledge it was your fault in not properly manipulating, instead of stuffing them with amalgam, and saying it was that black stuff that failed; that thing which was conceived from silver coin fillings and mercury, for the use of quack itinerants. Write to the *Missouri Journal* that you have graduated in amalgam filling, and, to use a California stock operator's phrase, "You sell it short."—*From Essay read before California State Dental Association.—Dental Register.*

---

